# Supplementary material for: Modeling Therapy-Driven Evolution of Glioblastoma with Patient-Derived Xenografts
Source: Cancers (Basel). 2022 Nov 9;14(22):5494. doi: 10.3390/cancers14225494 (PMC9688760; doi:10.3390/cancers14225494)
Supplement: Supplementary file 1 [file cancers-14-05494-s001.zip › mccord_MMR-PDX_MDPI_TableS1_FINAL.pdf]

Supplementary Table S1: Therapy Regimens for Derivative PDX

Derivative PDX	Parental PDX	RT, Days post-engraftment	TMZ Cycles: Days post-engraftment
m4052	GBM6	7-11	N/A
m4063	GBM6	7-11	N/A
m4066	GBM6	7-11	N/A
m6159	GBM12	7-11	N/A
m6161	GBM12	7-11	N/A
m3087	GBM43	7-11	N/A
m3098	GBM43	7-11	N/A
m4056	GBM6	N/A	7-11
m4057	GBM6	N/A	7-11
m4082	GBM6	N/A	7-11
m4883	GBM6	N/A	7-11
m2656	GBM12	N/A	7-11
m2657	GBM12	N/A	7-11
m3395	GBM6	7-11	7-11
m4051	GBM6	7-11	7-11
m2671	GBM12	7-11	7-11
m2685	GBM12	7-11	7-11
m2511	GBM12	7-11	#1: 7-11<br>#2: 45-49
m3378	GBM6	7-11	#1: 27-31<br>#2: 99-103<br>#3: 155-159
m4829	GBM12	N/A	#1: 27-31<br>#2: 48-52<br>#3: 55-59<br>#4: 97-101
m4834	GBM12	N/A	#1: 27-31<br>#2: 36-45<br>#3: 72-76<br>#4: 79-83<br>#5: 90
