# Supplementary material for: Modeling Therapy-Driven Evolution of Glioblastoma with Patient-Derived Xenografts
Source: Cancers (Basel). 2022 Nov 9;14(22):5494. doi: 10.3390/cancers14225494 (PMC9688760; doi:10.3390/cancers14225494)
Supplement: Supplementary file 1 [file cancers-14-05494-s001.zip › mccord_MMR-PDX_MDPI_TableS2_FINAL.pdf]

Supplementary Table S2: Most common mutated genes, by number of PDX with mutations

Gene	# of PDX with mutation	Derivatives with mutations
MACF1	10	m2511, m2656, m2685, m3378, m4056, m4057, m4066, m4829, m4834, m4883
TTN	9	m2657, m3098, m3378, m4057, m4082, m4829, m4834, m4883, m6161
DNAH5	9	m2656, m3378, m4051, m4056, m4057, m4063, m4082, m4834, m6161
SON	9	m2511, m2657, m2671, m3087, m3098, m4829, m4834, m6159, m6161
OR10H4	9	m2511, m2656, m2657, m2671, m2685, m4829, m4834, m6159, m6161
KLHL32	9	m2511, m2656, m2657, m2671, m2685, m4829, m4834, m6159, m6161
OSBPL5	8	m4051, m4052, m4056, m4057, m4063, m4082, m4829, m4883
ANKRD17	8	m2511, m2657, m2671, m3087, m3378, m4829, m4834, m6161
ISM1	8	m2511, m2656, m2657, m2671, m2685, m4829, m6159, m6161
USP34	8	m2511, m2657, m2671, m3087, m4829, m4834, m6159, m6161
CCAR2	8	m2511, m2656, m2657, m2671, m2685, m4829, m6159, m6161
CTCFL	8	m2511, m2656, m2657, m2671, m2685, m4051, m6159, m6161
CLN5	8	m2511, m2656, m2657, m2671, m2685, m3378, m6159, m6161
ACAN	7	m2657, m2671, m2685, m3378, m3395, m4051, m4834
DST	7	m2511, m2671, m3378, m4082, m4829, m4834, m4883
KMT2A	7	m2656, m2657, m2685, m3378, m4051, m4829, m6161
AHNAK2	7	m2671, m2685, m3378, m4051, m4056, m4829, m4834
KIAA1109	7	m2511, m2657, m2671, m2685, m4829, m4834, m6159
MUC5B	7	m2656, m2671, m2685, m4829, m4834, m4883, m6159
APC	7	m2511, m2656, m2657, m2671, m4051, m4834, m6159
KMT2C	7	m2671, m2685, m4051, m4057, m4829, m4834, m6159
NTRK3	7	m2511, m2657, m2671, m3087, m3098, m3378, m4834
CHD4	7	m2511, m2657, m4051, m4056, m4063, m4829, m4834
ZP2	7	m2511, m2656, m2657, m2671, m2685, m6159, m6161
ARID1B	7	m2656, m2657, m2671, m2685, m3378, m4834, m6161
RLN2	7	m2511, m2656, m2657, m2671, m2685, m6159, m6161
ZSWIM9	7	m2511, m2656, m2657, m2671, m2685, m6159, m6161
AHNAK	6	m2656, m2671, m2685, m3378, m4834, m4883
RNF213	6	m2657, m2671, m2685, m3378, m4063, m4834
BIRC6	6	m2511, m2671, m3087, m4829, m4834, m6161
FAT1	6	m2657, m2671, m2685, m3378, m4051, m4834
DNAH11	6	m2656, m3378, m4051, m4082, m4829, m4834
SZT2	6	m2656, m3378, m4051, m4057, m4066, m4834
MGA	6	m2671, m2685, m3087, m3378, m4051, m4834
COL12A1	6	m2656, m2657, m2671, m2685, m3098, m4834
NCOR1	6	m2657, m2671, m3378, m4057, m4834, m6159
PHF3	6	m2511, m2657, m2671, m4834, m6159, m6161
MSH6	6	m2671, m3378, m3395, m4051, m4829, m4834
UBN2	6	m2511, m2656, m2657, m2671, m4056, m4082
BRD8	6	m2511, m2685, m4052, m4834, m6159, m6161
NRP2	6	m2657, m2671, m2685, m4829, m4834, m6159
BBS9	6	m2656, m2657, m2671, m3378, m4829, m4834
ZNF724	6	m2511, m2656, m2657, m2671, m6159, m6161
KHDC4	6	m2511, m2657, m2671, m2685, m3378, m6159
HMGXB4	6	m2656, m2657, m2671, m2685, m4051, m4834

URI1	6	m2656, m2657, m2671, m4829, m6159, m6161
CYP4F2	6	m2671, m2685, m4829, m4834, m6159, m6161
OR2B11	6	m2511, m2656, m2671, m2685, m6159, m6161
AQP12B	6	m2511, m2657, m2671, m2685, m4829, m4834
OBSCN	5	m2656, m2671, m3378, m4829, m4834
SYNE1	5	m2671, m3378, m4057, m4829, m4834
MDN1	5	m2657, m2671, m2685, m4829, m4834
FCGBP	5	m2511, m3087, m4051, m4829, m4834
ABCA13	5	m2671, m3378, m4057, m4829, m4834
DNHD1	5	m2511, m2656, m4051, m4829, m4834
VPS13D	5	m2657, m2671, m2685, m4829, m4834
ANK3	5	m2671, m3378, m4056, m4829, m4834
ALMS1	5	m2671, m3378, m4057, m4829, m4834
DNAH3	5	m2657, m2671, m2685, m4829, m4834
CMYA5	5	m2685, m3378, m4051, m4057, m4829
TRRAP	5	m2511, m3378, m4056, m4057, m4829
CSMD3	5	m2671, m4829, m4834, m6159, m6161
SMG1	5	m2685, m3378, m4082, m4829, m4834
DCHS2	5	m3378, m4056, m4057, m4829, m4834
LAMA1	5	m2671, m3378, m4829, m4834, m4883
FRYL	5	m2656, m2671, m3378, m4829, m4834
LRBA	5	m2657, m2685, m3378, m4834, m6159
CREBBP	5	m3378, m4051, m4829, m4834, m6161
CACNA1G	5	m2511, m2657, m2671, m4829, m4834
PCNX1	5	m2511, m3087, m3378, m4057, m4834
CACNA1C	5	m2511, m3378, m4082, m4829, m4834
CACNA1D	5	m2511, m4829, m4834, m6159, m6161
BAZ2B	5	m2657, m2685, m4051, m4056, m4834
RP1	5	m2657, m2671, m2685, m4057, m4834
ATAD5	5	m2657, m2671, m4051, m4057, m4834
TNRC6B	5	m2511, m2657, m4834, m6159, m6161
C2CD6	5	m2657, m2671, m2685, m3378, m4834
ARHGEF4	5	m2511, m2671, m3378, m4051, m4834
MBD5	5	m2657, m2671, m4829, m4834, m6159
SOGA1	5	m3378, m4051, m4066, m4829, m4834
MROH1	5	m2511, m3378, m4051, m4829, m4834
C4orf50	5	m2656, m2671, m4829, m4834, m6159
KDM6A	5	m2657, m2685, m3098, m4834, m6159
TNR	5	m2511, m2671, m4051, m4834, m6161
SPAG9	5	m2657, m2671, m2685, m3378, m4063
SORBS1	5	m2511, m2657, m4829, m4834, m6159
KCNMA1	5	m2511, m2671, m3087, m4051, m6159
POM121	5	m2511, m2685, m3378, m4829, m4883
CSPP1	5	m2657, m2671, m2685, m3378, m4834
MAMDC4	5	m2511, m4829, m4834, m4883, m6161
SMG7	5	m2657, m2685, m3087, m3378, m6159
COL6A1	5	m2671, m3378, m4051, m4829, m4834
PPP6R2	5	m2511, m2657, m2685, m4834, m6161
MEIOC	5	m2657, m2671, m2685, m3098, m4057
WWP1	5	m2671, m2685, m4829, m4834, m6161
PAXBP1	5	m3378, m4051, m4829, m6159, m6161
MATR3	5	m2511, m4082, m4834, m6159, m6161
ZBTB10	5	m2511, m4052, m4829, m6159, m6161

PPP4R3A	5	m2671, m2685, m3378, m4057, m6161
VWA5A	5	m2657, m2671, m2685, m3378, m4829
CNOT3	5	m2511, m2657, m4829, m6159, m6161
NOA1	5	m2656, m2657, m2671, m2685, m4883
TRAF7	5	m2657, m2671, m2685, m3378, m4051
ANO10	5	m2656, m2657, m2671, m2685, m3378
AVL9	5	m2657, m2671, m2685, m4056, m4834
BRD9	5	m2671, m2685, m4829, m4834, m6161
EBF3	5	m2657, m2671, m4063, m4834, m6161
ITPRIPL1	5	m2656, m2657, m2671, m2685, m3395
CPNE4	5	m2656, m2657, m2671, m2685, m4829
PTBP3	5	m2671, m3087, m4834, m6159, m6161
MIER2	5	m4056, m4057, m4063, m4082, m4829
LYN	5	m2511, m2657, m2671, m6159, m6161
GRSF1	5	m2657, m2671, m2685, m4063, m4834
FUZ	5	m2657, m2671, m2685, m4829, m4834
PILRA	5	m2511, m2656, m2671, m4829, m6159
