# Supplementary figures and images for: Modeling Therapy-Driven Evolution of Glioblastoma with Patient-Derived Xenografts
Source: Cancers (Basel). 2022 Nov 9;14(22):5494. doi: 10.3390/cancers14225494 (PMC9688760; doi:10.3390/cancers14225494)

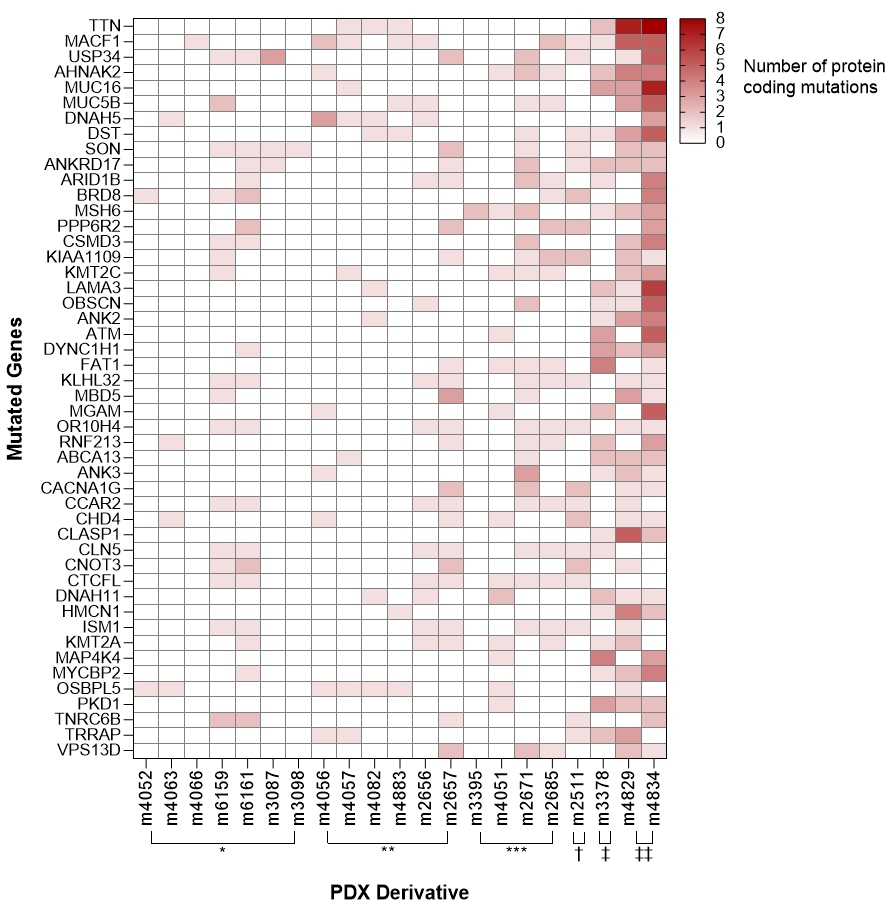

Supplement: Supplementary file 1 [file cancers-14-05494-s001.zip › Figure_S1_FINAL.png]

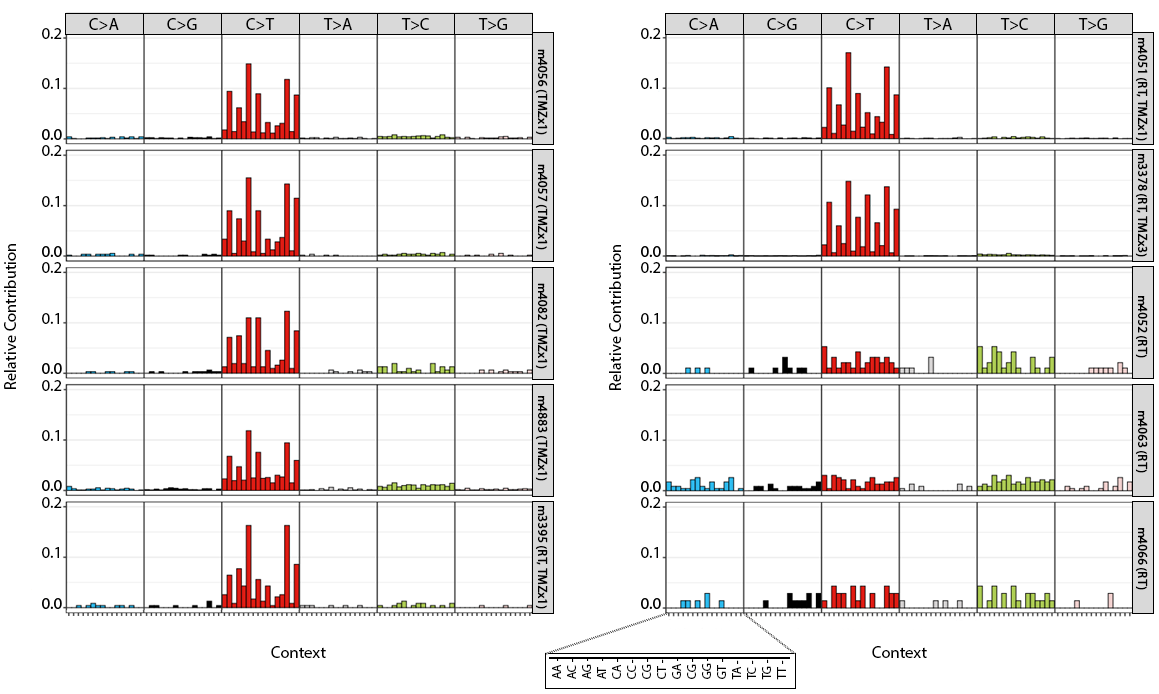

Supplement: Supplementary file 1 [file cancers-14-05494-s001.zip › Figure_S2_FINAL.png]

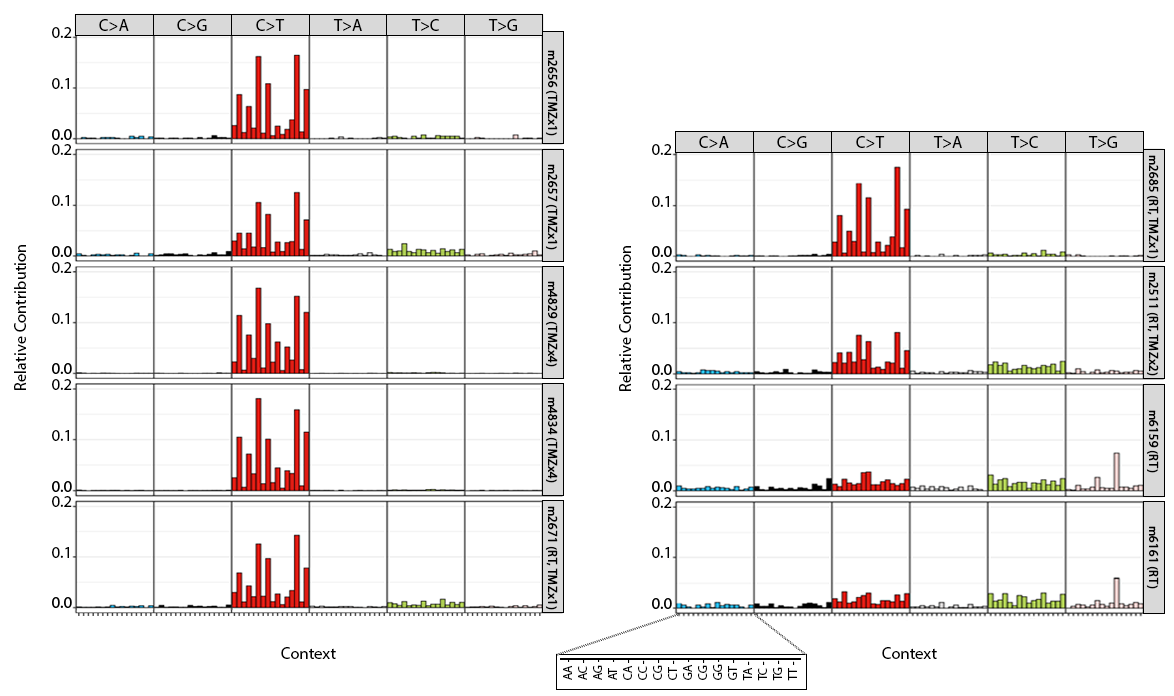

Supplement: Supplementary file 1 [file cancers-14-05494-s001.zip › Figure_S3_FINAL.png]

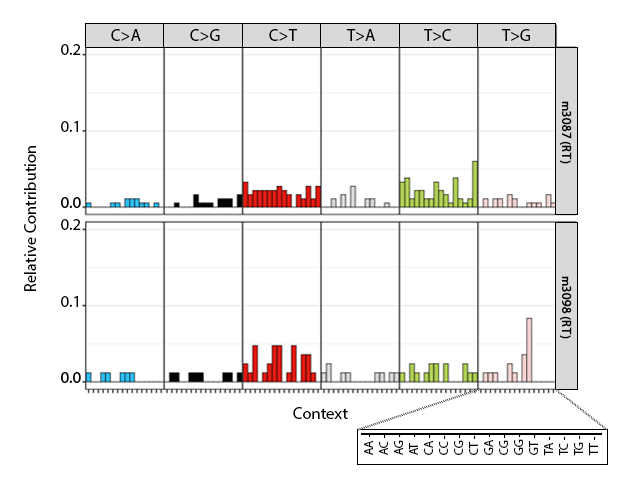

Supplement: Supplementary file 1 [file cancers-14-05494-s001.zip › Figure_S4_FINAL.png]
